# Regulation of Renin Expression by Β1-Integrin in As4.1 Juxtaglomerular Line Cells

**DOI:** 10.3390/biomedicines11020501

**Published:** 2023-02-09

**Authors:** Nobumichi Saito, Masao Toyoda, Masumi Kondo, Makiko Abe, Noriyuki Sanechika, Moritsugu Kimura, Kaichiro Sawada, Masafumi Fukagawa

**Affiliations:** Division of Nephrology, Endocrinology and Metabolism, Department of Medicine, Tokai University School of Medicine, 143 Shimokasuya, Isehara 259-1193, Kanagawa, Japan

**Keywords:** diabetic nephropathy, hypertension, renin, β1-integrin, phosphorylation

## Abstract

(1) Background: Renal dysfunction and hypertension are mutually aggravating factors; however, the details of their interaction remain unclear. In a study using renal tissue from diabetic rats, we found that β1-integrin, a cell-substrate adhesion molecule, is specifically phosphorylated in juxtaglomerular cells that secrete renin, a blood pressure regulator. (2) Methods: A mouse juxtaglomerular cell line (As4.1 cells) was used for the following experiments: drug-induced promotion of β1-integrin phosphorylation/dephosphorylation; knockdown of β1-integrin and the cell adhesion molecule connexin-40 (a candidate for the main body of baroreceptor); and pressurization to atmospheric pressure + 100 mmHg. culture in hypotonic liquid medium. The expression of renin under these conditions was measured by qRT-PCR. (3) Results: Phosphorylation of β1-integrin suppressed the expression of renin, while dephosphorylation conversely promoted it. β1-integrin and connexin-40 knockdown both promoted the expression of renin. Pneumatic pressurization and hypotonic medium culture both decreased the expression of renin, which was restored by the knockdown of β1-integrin. (4) Conclusions: β1-integrin plays an inhibitory role in the regulation of the expression of renin, which may be controlled by phosphorylation and dephosphorylation. It is hypothesized that β1-integrin and other adhesion factors regulate the expression of renin by altering the sensitivity of baroreceptors on the plasma membrane.

## 1. Introduction

Hypertension induced by diabetes mellitus (DM) promotes diabetic nephropathy (DN) or diabetic kidney disease (DKD) [1,2,3,4,5,6]. DM impairs the coordination of the myogenic response [7,8] and tubuloglomerular feedback (TGF) [9], resulting in loss of control over the vascular response of the afferent arterioles, which induces the expansion of the afferent arterioles and consequent hyperfiltration and capillary hypertension in the glomeruli [3]. These effects induce renal oxidative stress and inflammation, which is thought to be the cause of DN [4]. Activation of the renin-angiotensin (RA) system [10], in which the increased expression of endothelin-1 (ET-1) [11,12] and reactive oxygen species [13,14] and decreased nitric oxide (NO) occurs [15,16], increases the blood pressure and worsens the renal function.

Renin, the rate-limiting enzyme of the RA system, is secreted by juxtaglomerular cells (JGC), and its expression is regulated by two major mechanisms. One is regulation by regulatory signals from outside the JGC, such as the renal sympathetic nerves, which sense blood flow and blood pressure, and maculadensa cells that sense glomerular filtration rate, and the other is regulation by the JGC themselves, which sense blood pressure in the afferent arterioles [17,18,19,20,21]. The latter mechanism may contribute significantly to the onset and progression of DN, but its details, including the main body of baroreceptor molecules, have not been elucidated.

Connexin, an intercellular adhesion molecule, has been reported as a factor involved in blood-pressure-sensing in JGC. Connexin-40 (CX40) is abundantly expressed in JGC, and its knockout mice show hypertension due to the inability to inhibit renin secretion; this has been considered one of the candidates for the main body of the baroreceptor [22]. However, it was recently revealed that β1-integrin, a substrate-cell adhesion molecule, is also involved in JGC pressure-sensing. It has been reported that β1-integrin knockout mice have greatly reduced renin secretion, contrary to that of CX40 [23,24]. Considering these reports, it is interesting that these molecules, both of which have cell adhesion functions, are involved in pressure-sensing.

Unlike CX40, the expression of which is concentrated in JGCs, β1-integrin is also expressed in many other cell types in the kidney, e.g., mesangium and podocytes in glomeruli, Bowman’s capsule, proximal tubule epithelial cells, distal tubular cells, and cells in collecting ducts [25], making it an unlikely candidate for a JGC-specific baroreceptor molecule. Rather, β1-integrin may function as an essential factor in the baroreceptor-containing blood-pressure-sensing mechanisms.

We have discovered and reported the phosphorylation of two juxtaglomerular apparatus (JGA)-specific β1-integrins: the JGC-specific phosphorylation of the threonine 788/789 site (Thr788/789) and the maculadensa-specific phosphorylation of the serine 785 site (Ser785) in β1-integrin. In a previous study of DN model rats produced by the administration of streptozotocin [26], phosphorylation of the β1-integrin Thr788/789 continued to gradually decrease after the onset of DN, but reversed and began to increase from 2 months post-onset and recovered to almost the level at the onset by 6 months post-onset. These changes of Thr788/789 phosphorylation were negatively correlated with the renin expression in JGC during the progression of DN, suggesting a possibility that phosphorylation of this site may be involved in the regulation of renin production by β1-integrin [26].

In the present study, we confirm that β1-integrin regulates renin production through phosphorylation/dephosphorylation and examine how β1-integrin affects pressure sensing systems in JGC.

## 2. Materials and Methods

### 2.1. Cell Culture

The established mouse juxtaglomerular cell-line As4.1 [27] was purchased from ATCC (CRL-2193, Manassas, VA, USA) and cultured with DMEM medium (ATCC) containing 50 U/mL penicillin, 50 mg/mL streptomycin and 10% fetal bovine serum in a 5% CO_2_ atmosphere at 37 °C. Transforming growth factor-β1 (1 ng/mL; TGF-β1; R&D Systems), phorbol 12-myristate 13-acetate (50 ng/mL; PMA; R&D Systems, Minneapolis, MN, USA), and bisindolylmaleimide (2 μM; BIM-1; R&D Systems) were excipients for cultures.

### 2.2. Gene Knockdown Experiments

For knockdown experiments, Stealth RNAi siRNAs for CX40 (MSS236624; ThermoFisher, Waltham, MA, USA) or β1-integrin (MSS205553; ThermoFisher) were mixed with RNAiMax transfection reagent (ThermoFisher) according to manuals of these reagents. Then, the mixtures were added to the cell cultures with 50 nM of the final concentration of each siRNA. After 24 h of transfection, culture medium was replaced with fresh medium without siRNA and incubated for more 24 h. At the time of harvest of cells, cell numbers of a part of the cultures were counted, and survival rates were checked. 

Some cell cultures were subjected to pressurization. Cells were cultured in an airtight acrylic container placed in a 5% CO_2_ atmosphere at 37 °C, and 100 mmHg of air pressure was added by a manual pump connected to the container with a barometer. The air pressure was maintained for 24 h. In another experiment, cells were cultured in hypotonic medium with a lower salt content in comparison to normal medium. The osmotic pressure was measured by osmometer (Osmomat, Gonotech, Berlin, Germany). 

### 2.3. Quantitative Real-Time Polymerase Chain Reaction (qRT-PCR)

Total RNA was extracted from cultured cells using RNA purification kit (RNaqueous; ThermoFisher). One μg of total RNA of each sample was converted to cDNA by SuperScript IV reverse transcriptase (ThermoFisher). qRT-PCR was performed as described previously [28]. The reaction mixture containing primers and probes for mouse renin (Assay ID: Mm02342887_mH), β1-integrin (Assay ID: Mm01253230_m1), CX40 (Gja5; Assay ID: Mm1265686_m1) and endogenous controls (18S ribosomal RNA or β-actin) was prepared with TaqMan Gene Expression Assays (ThermoFisher). PCR was performed by ABI Quantstudio 3 (ThermoFisher). Data were analyzed by the comparative Ct method. Expression levels of target mRNAs were compared to endogenous controls and standardized with negative controls.

### 2.4. Western Blotting

Cell lysates were prepared with xTractor lysation buffer (Takara Bio, Shiga, Japan) containing phosphatase inhibitor cocktail (Nakarai Tesque, Kyoto, Japan), ProteoGuard protease inhibitor (Takara Bio), and Cryonase nuclease (Takara Bio). Each 25 μg of total protein were loaded on NuPAGE 4–12% Bis-Tris gel (ThermoFisher) and Sodium dodecyl sulfate-polyacrylamide gel electrophoresis (SDS-PAGE) was performed. Then, proteins were blotted on a PVDF membrane with the iBlot dry blotting system (ThermoFisher). Blocking was performed with 5% bovine-albumin in Tris-buffered saline (TBS), and the PVDF membrane was reacted with the rabbit polyclonal antibodies for Thr788/789-phospho-integrin β1 (PA5-36779; ThermoFisher), washed with TBS with 0.05% Tween 20 overnight at 4 °C. After washing with TBS—0.05% Tween20, the membrane was treated with horseradish peroxidase-conjugated anti-rabbit IgG antibody. After washing with TBS—0.05% Tween20, the reactions were visualized with diaminobenzidine (DAB).

### 2.5. Statistical Analyses

Results represent the mean (±standard deviation, SD) of at least four samples. Comparisons between two groups were performed using Student’s *t*-test. *p* values of <0.05 were considered statistically significant. 

## 3. Results

### 3.1. Activation/Inhibition of Protein Kinase Cε Reproduced the Negative Correlation between Threonine-788/789-Phosphorylated Β1-Integrin and the Renin Expression In Vitro

In our previous study, a negatively correlated relationship between renin expression and Thr788/789 phosphorylation of β1-integrin in JGC was detected in the kidneys of STZ rats (26); thus, as the next step, the relationship between β1-integrin phosphorylation/dephosphorylation and renin production was re-examined with cultured cells in vitro. A line of mouse JGC, As4.1, was used to examine the changes in renin expression after the addition of PMA/BIM-1, an activator/inhibitor of PKCε, which is a PKC subtype that phosphorylates Thr788/789 of β1-integrin [29]. As TGF-β1 is reported to promote the Thr788/789 phosphorylation of β1-integrin specifically [30], we also examined the effect of the addition of TGF-β1 to the culture. 

**Figure 1:**
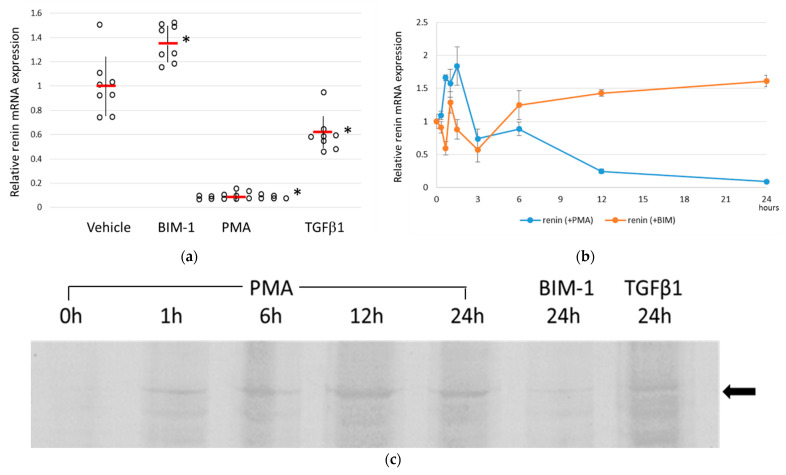
Phosphorylation/dephosphorylation of Thr788/789 of β1-integrin effect on the renin expression in a juxtaglomerular cell line As4.1. (**a**) Two μM BIM-1, 50 ng/mL PMA and 1 ng/mL TGF-β1 were added to the culture of As4.1 cells for 24 h. The renin expression levels were examined by qRT-PCR, and the expression levels relative to control (vehicle) were represented by scatter plot with the average (red bars) and SD (black vertical lines). Significant differences (*p* < 0.05) from the control were indicated with asterisks. (**b**) The renin mRNA expression in As4.1 cells cultured with 2 μM BIM-1 (orange) or 50 ng/mL PMA (blue) in 24 h time-course measured by qRT-PCR. Each point was the average of at least 4 samples. (**c**) Detection of phosphorylation/dephosphorylation of Thr788/789 of β1-integrin in As4.1 cells. Cells were cultured with 50 ng/mL PMA (for 1, 6, 12, and 24 h, 2 μM BIM-1 for 24 h or 1 ng/mL TGF-β1 for 24 h. Phosphorylated phosphor-Thr788/789 inβ1-integrin was detected by a specific antibody. The side arrow represents the position of phospho-Thr788/789 β1-integrin bands.

The results showed that renin expression in As4.1 cells at 24 h after the addition of the drug was significantly inhibited by PKC activation by PMA and, conversely, renin expression was significantly enhanced when PKC was inhibited by BIM-1, confirming a negative relationship between renin expression and phosphorylation of β1-integrin at Thr788/789, similar to that observed in vivo (Figure 1a). TGF-β1 also significantly inhibited renin expression and supported the relationship. When the changes in renin expression up to 24 h after PMA/BIM-1 treatment were monitored, it was found that the renin expression showed severe up and down in the early period up to three hours after drug treatment, but thereafter a gradual suppression and steady increase until the end of the experiment were observed (Figure 1b). Western blotting for phosphorylated Thr788/789 of β1-integrin showed compatible results with the above findings, wherein the promotion of phosphorylation by PMA occurred 3 h after the drug initiation and was sustained for 24 h. BIM-1 inhibited the phosphorylation of these sites, and TGF-β1 accelerated the phosphorylation of these sites, but not as much as PMA (Figure 1c).

### 3.2. Knockdown of the Β1-Integrin Expression Results in the Uncontrolled Expression of Renin, as Does Knockdown of the CX40 Expression

**Figure 2:**
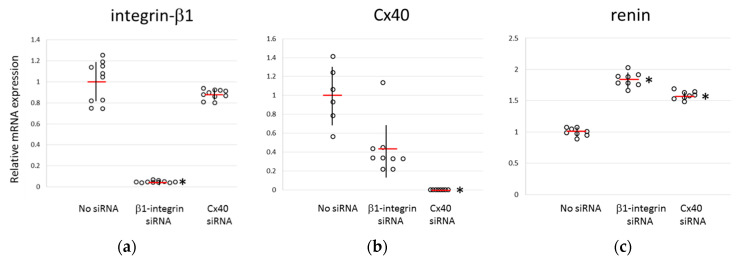
Effects of knockdown of β1-integrin or Cx40 on the renin expression in a juxtaglomerular cell line (As4.1 cells). Gene knockdown was performed by culturing As4.1 cells with siRNA for β1-integrin or siRNA for Cx40 for 24 h. Vehicle was added to the control culture (no siRNA). The expression of β1-integrin (**a**), CX40 (**b**) and renin (**c**) was quantitated by qRT-PCR. The expression relative to individual controls (no siRNA) is represented by scatter plot with the average (red bars) and SD (black vertical lines). Asterisks indicate a significant difference (*p* < 0.05) from the control (no siRNA).

Since PMA and BIM have a broad spectrum of effects across several PKC subtypes, the possibility that the changes in renin expression shown in Figure 1 were overlapped results of other phosphorylation pathways with the phosphorylation of Thr788/789 in β1-integrin by PKCε was not excluded (about the effects of these drugs to canonical target, (see Supplementary Figure S1). In order to explain the function of β1-integrin on renin expression, knockdown of β1-integrin in As4.1 cells was performed, and renin expression was examined. Results in Figure 2 showed that the knockdown of β1-integrin in As4.1 cells enhanced renin expression level upto about twice of that in control. Renin expression was also increased by knockdown of Cx40, a putative baroreceptor, to the same level, suggesting that these renin expression levels represent an uncontrollable state of renin production by JGC (Figure 2).

### 3.3. The Renin Gene Expression under Air Pressure with/without Β1-Integrin Knockdown

**Figure 3:**
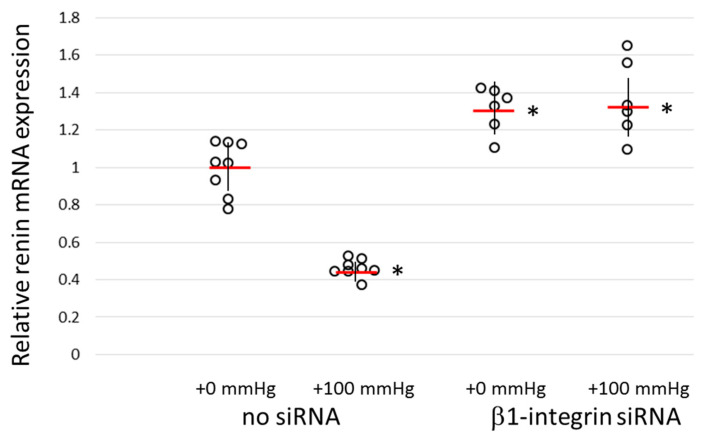
Effect of air pressurization on the renin expression in a juxtaglomerular cell line (As4.1 cells). As4.1 cells were cultured under atmospheric pressure +100 mmHg for 24 h and the renin expression was examined by qRT-PCR (+100 mmHg). Control cells were cultured under normal atmospheric pressure (+0 mmHg). Some cultures were pressurized after β1-integrin knockdown with siRNA (β1-integrin siRNA). Data were represented by scatter plot with the average (red bars) and SD (black vertical lines). Asterisks indicate a significant difference (*p* < 0.05) from the control (+0 mmHg, no siRNA).

The knockdown experiment confirmed that β1-integrin is required for the regulation of the renin gene expression in cultured JGC. We investigated whether these JGC can actually sense pressure to cells. As shown in Figure 3, the renin gene expression was significantly reduced to approximately half of that of the non-pressurized control by applying 100 mmHg of pressure to normal atmospheric pressure; however, when β1-integrin was knocked down in advance, no reduction in renin expression was observed. These results indicate that β1-integrin is required for JGC to sense pressure and suppress the expression of renin.

### 3.4. The Renin Gene Expression in Hypotonic Medium with/without Β1-Integrin Knockdown

**Figure 4:**
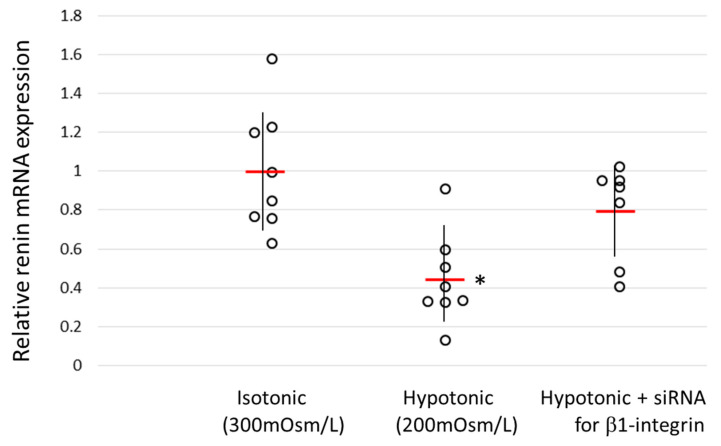
Effect of hypotonic medium on renin expression in a juxtaglomerular cell line (As4.1 cells). As4.1 cells were cultured in hypotonic medium (200 mOsm/L). After 24 h, the renin expression was examined by qRT-PCR. Control cells were cultured in isotonic medium (300 mOsm/L). Some were cultured in hypotonic medium after β1-integrin knockdown with siRNA (Hypotonic + siRNA forβ1-integrin). Data were represented by scatter plot with average (red bars) and SD (black vertical lines). Asterisks indicate significant differences (*p* < 0.05) from the control (Isotonic).

The above results confirm that β1-integrin is involved in the sensing of pressure by JGC. Since β1-integrin is a substrate-cell adhesion factor, its reduction by knockdown may reduce plasma membrane tension. To investigate how changes in the membrane tension affect the regulation of the renin expression by β1-integrin, we measured the renin expression when JGC were cultured in hypotonic medium. As shown in Figure 4, the renin gene expression in JGC was significantly decreased in hypotonic medium, but the degree of decrease was attenuated and the significant difference was canceled when β1-integrin was knocked down beforehand. These results indicate that membrane tension of JGC affects the suppression of the expression of renin by β1-integrin, since the expression of several representative genes in AS4.1 cells was unchanged in hypotonic medium (see Supplementary Figure S2).

## 4. Discussion

In our previous paper, we reported that β1-integrin is phosphorylated in a cell type-specific manner in JGA and that the degree of phosphorylation is associated with the progression of DN, especially renin production, in STZ rats [26]. JGC-specific β1-integrin phosphorylation occurs at the Thr788/789 sites and the amount was negatively correlated with renin production in the kidneys of DN rats. In this study, we investigated the relationship between β1-integrin with/without phosphorylation and the renin expression from the viewpoint of pressure-sensing by performing in vitro experiments using JGC capable of renin production.

**Figure 5:**
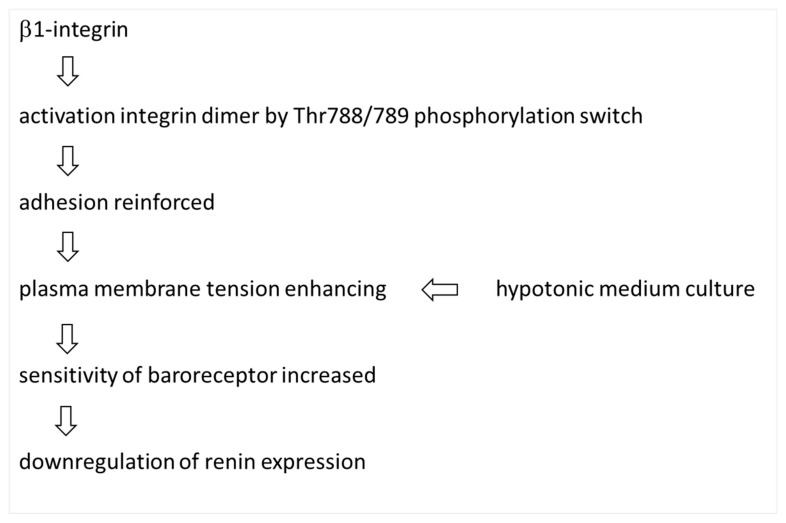
Schematic illustration of the hypothesized mechanisms underlying the regulation of renin by phosphorylated β1-integrin.

From these results, it is possible to infer a mechanism by which β1-integrin is involved in cellular baroreceptor sensing (Figure 5). That is, β1-integrin may affect the sensitivity of baroreceptors on the plasma membrane by modulating the strength of substrate-cell adhesion, resulting in changes in plasma membrane tension and other properties. The Thr788/789 phosphorylation of β1-integrin has been reported to affect cell adhesion and motility [29,30,31], and recent reports indicate that this phosphorylation site of β1-integrin functions as a phosphorylation switch that activates/inactivates the integrin dimer function, thereby increasing or decreasing cell adhesion [32]. In JGC, the β1-integrin phosphorylation switch may contribute to the regulation of the expression of renin, a specific function of JGC.

Recently, other research groups have reported that β1-integrin is required for the regulation of renin production. In these papers, they generated genetically engineered mice in which the β1-integrin gene was knocked out specifically in cells expressing the renin gene, and showed that in these knockout mice, the production of renin does not increase with decreasing blood pressure, and that in cultures of smooth muscle cells of afferent arterioles stimulated to produce renin, the production of renin is reduced by pressure and cell stretching [23,24]. Knockout of β1-integrin results in abnormal kidney development and renin-producing cells undergo apoptosis, so renin-producing capacity is low as well. The knockout mice showed that β1-integrin is essential for the survival of renin-producing cells during development, but may not be able to carry out the function of β1-integrin in the normal kidney.

In our experiments, the knockdown of β1-integrin on As4.1 cells enhanced the expression of renin. Since there was no mass death of cultured cells upon knockdown of β1-integrin, knockdown in culture did not affect the survival of renin-producing cells, but rather the regulatory function of renin production via pressure-sensing. The knockdown of CX40 increased the expression of renin by approximately the same amount. CX40 knockout mice have been reported to be hypertensive [22], which is consistent with this result. Thus, it is possible that the basic function of β1-integrin and CX40 is to suppress the expression of renin. The phosphorylation switch concept, wherein β1-integrin is functionally activated by the phosphorylation of Thr788/789 [32] is consistent with the fact that the amount of phosphorylation at this site is negatively correlated with the renin expression and that β1-integrin knockdown results in the increased expression of renin.

When As4.1 cells were incubated under an extra 100 mmHg of air pressure for 24 h, the renin expression was reduced by half. This result is the same as that obtained by Watanabe et al. in their experiments with renin-producing cells [24]. When As4.1 cells with β1-integrin knockdown were subjected to 100 mmHg of extra air pressure, their renin expression was unchanged from that of non-pressurized cells. This result also indicates that loss of β1-integrin results in the inability to suppress the production of renin.

In the present experiment, renin mRNA often changed in the range of 1.5 to 2 times that of the control, but it is not clear whether changes of this magnitude are effective in regulating blood pressure. However, since the normal blood renin concentration in humans varies within a relatively narrow range of 0.3 to 3 ng/mL/h, it is possible that small changes in mRNA levels could cause large changes in blood pressure. The short half-life of renin (30–120 min) also suggests a large influence of changes in mRNA levels. β1-integrin phosphorylation may function to suppress such renin mRNA expression through subtle regulation.

The baroreceptors of JGC are located on the plasma membrane, but the details of how they sense increases or decreases in pressure are not clear. The renin expression was significantly reduced when As4.1 cells were swollen in hypotonic medium. Watanabe et al. also reported that the expression of renin was reduced when renin-producing cells were stretched [24]. These facts suggest that the sensitivity of baroreceptors is sensitized by an increase in cell membrane tension. In other words, the baroreceptors sensitized by increased plasma membrane tension overestimated the pressure exerted on the cells and reduced the expression of renin, even though the pressure on the cells did not change. The decrease in the production of renin in the hypotonic medium tended to be moderated when the expression of β1-integrin was knocked down beforehand, confirming the inhibitory effect of β1-integrin on the expression of renin. In other words, the loss of β1-integrin may have offset the sensitization of baroreceptors by the hypotonic medium.

A possible explanation for how cell adhesion factors, such as β1-integrin and CX40, regulate baroreceptor sensing is that changes in plasma membrane tension due to increased or decreased adhesion alter the sensitivity of baroreceptors on the plasma membrane (Figure 5). Since the knockdown of β1-integrin affects the renin gene expression, signals from baroreceptors should reach the nucleus to turn renin genes on and off. Watanabe et al. have shown that β1-integrin regulates lamin, a nuclear membrane lining protein, through reorganization of actin filaments, and that lamin, which is also known to be involved in transcription, turns renin genes on and off [24]. Although it is unclear whether β1-integrin and CX40 themselves or other molecules are the main body of the baroreceptor, present report suggests that a detailed understanding of the molecular identity of the JGC baroreceptor and the regulatory mechanism of renin expression is needed to stop the reciprocal vicious cycle of diabetes and hypertension.

As mentioned previously, DM impairs myogenic responses [7,8] and tubuloglomerular feedback (TGF) [9], functions that autoregulate renal blood flow, causing import arteries to dilate, leading to glomerular hyperfiltration and glomerular capillary hypertension [3]. At this time, the cell membrane of JGCs attached to the basement membrane of imported arterioles should also stretch along with the basement membrane, so renin production should decrease according to the experimental results of Watanabe and our culture system, but in reality, hypertension is often maintained. In this condition, the suppressive mechanism of renin expression is thought to be disrupted, and decreased expression of Cx40 and β1-integrin in JGC, as well as decreased phosphorylation of β1-integrin, may be the major factors.

Elucidating the regulatory mechanism of renin expression in JGC may contribute to the treatment of hypertension, a condition in which the normal function of the myogenic response and TGF system is lost. The culture system used in this study will be very useful for this purpose. The cell culture system can create an environment that eliminates complex interactions between organelles. As mentioned earlier, it is well known that renin production in JGCs is affected by numerous factors external to JGCs, but perfusion experiments using isolated glomeruli have revealed that JGCs themselves regulate renin expression by sensing blood flow and blood pressure [20,21]. However, the regulatory mechanism has been difficult to analyze in previous studies at the individual and tissue levels, and no significant progress has been made in subsequent studies. Investigations using cell culture systems are expected to advance our understanding of the mechanisms linking JGC pressure sensing and renin expression, which are important for blood pressure regulation.

On the other hand, however, our culture system may not be effective in detecting glycotoxicity: As4.1 cells are juxtaglomerular cells immortalized by transfection with the SV40T gene, a known oncogene. In general, cancer cells are known to have renewed glycolytic metabolism due to the Warburg effect, and their proliferation becomes more active when the glucose concentration in the medium is increased [33,34]. In fact, As4.1 cells were cultured in a high glucose concentration medium of 4.5 g/L, which is higher than the glucose concentration of 1 g/L in the normal medium, suggesting that they are tolerant to high glucose concentration. Analysis of the effects of the diabetic environment on renin production and blood pressure regulation would require the use of other suitable cell culture systems.

## 5. Conclusions

In the As4.1 JGC cell line, phosphorylation of β1-integrin at Thr788/789 suppressed renin expression, while dephosphorylation increased renin expression, indicating that renin expression may be regulated by phosphorylation/dephosphorylation of the same site. In addition, knockdown of β1-integrin in the same culture system enhanced renin expression, which was equivalent to the effect of knockdown of Cx40, a predicted baroreceptor. Renin expression in the same cells was decreased by both atmospheric pressure and hypotonic medium culture, but prior knockdown of β1-integrin suppressed the decrease in renin expression. These results indicate that β1-integrin is suppressively involved in the regulation of renin expression and that phosphorylation/dephosphorylation of its Thr788/789 site can subtly regulate renin expression. β1-integrin is an important factor in the regulation of renin production by JGC pressure sensing. It is suggested that changes in substrate adhesion by phosphorylation/dephosphorylation regulate renin expression by altering the sensitivity of the baroreceptors on the plasma membrane of JGCs. The JGC culture system may be useful for a detailed analysis of this mechanism.

## Data Availability

Not applicable.

## Supplementary Materials

The following supporting information can be downloaded at: https://www.mdpi.com/article/10.3390/biomedicines11020501/s1, Figure S1: Effects of phosphorylation/dephosphorylation-drugs on canonical targets expressed in a juxtaglomerular cell line (As4.1 cells); Figure S2: Effect of hypotonic medium on gene expressions in a juxtaglomerular cell line (As4.1 cells).
