# Supplementary material for: Regulation of Renin Expression by Β1-Integrin in As4.1 Juxtaglomerular Line Cells
Source: Biomedicines. 2023 Feb 9;11(2):501. doi: 10.3390/biomedicines11020501 (PMC9953579; doi:10.3390/biomedicines11020501)
Supplement: Supplementary file 1 [file biomedicines-11-00501-s001.zip › Fig_S2.pdf]

Supplementary

Regulation of renin expression by β1-integrin in As4.1 juxtaglomerular line cells

Nobumichi Saito, Masao Toyoda*, Masumi Kondo, Makiko Abe, Noriyuki Sanechika, Moritsugu Kimura, Kaichiro Sawada, and Masafumi Fukagawa

Division of Nephrology, Endocrinology and Metabolism, Department of Medicine, Tokai University School of Medicine, 143 Shimokasuya, Isehara, Kanagawa, 259-1193, Japan

* Correspondence: m-toyoda@is.icc.u-tokai.ac.jp; Tel.: +81-463-93-1121(ext. 2490)

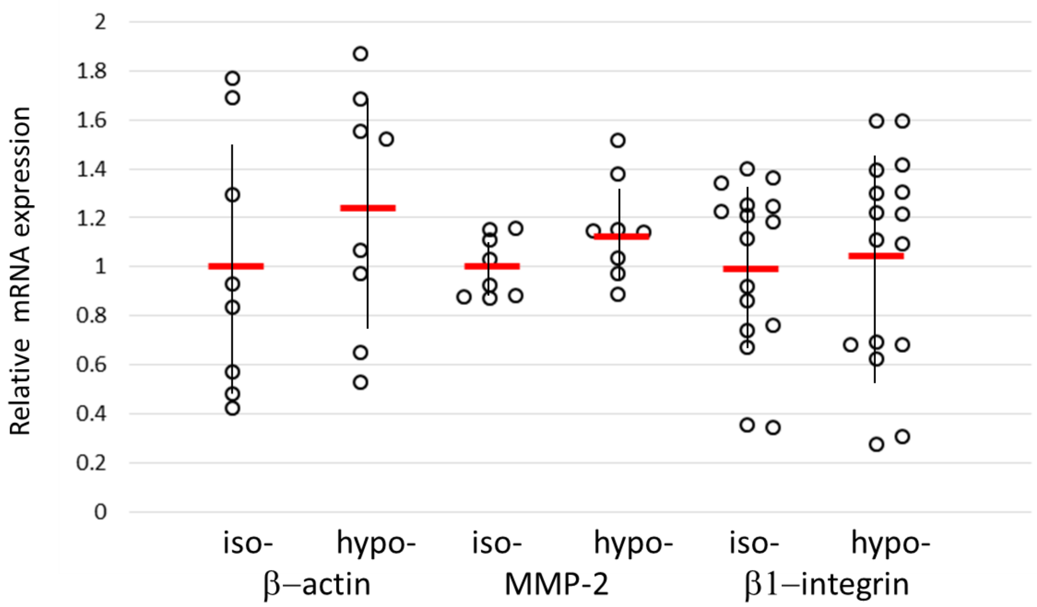

Figure S2. **Effect of hypotonic medium on gene expressions in a juxtaglomerular cell line (As4.1 cells).** As4.1 cells were cultured in hypotonic medium (hypo- ; 200 mOsm/L). After 24 hours, expressions of β-actin, Matrix metalloproteinase 2 (MMP-2) and β1-integrin were examined by qRT-PCR with TaqMan Gene Expression Assays (ThermoFisher), containing primers and probes for mouse β-actin, MMP-2 (Assay ID: Mm00439498_m1), β1-integrin and 18S ribosomal RNA as an endogenous control. Control cells were cultured in isotonic medium (iso- ; 300 mOsm/L). Data

were represented by scatter plot with average (red bars) and S.D. (black vertical lines). No significant differences ($p<0.05$) was observed between iso- and hypo-.
