# Supplementary material for: Regulation of Renin Expression by Β1-Integrin in As4.1 Juxtaglomerular Line Cells
Source: Biomedicines. 2023 Feb 9;11(2):501. doi: 10.3390/biomedicines11020501 (PMC9953579; doi:10.3390/biomedicines11020501)
Supplement: Supplementary file 1 [file biomedicines-11-00501-s001.zip › Fig_S1.pdf]

Supplementary

Regulation of renin expression by β1-integrin in As4.1 juxtaglomerular line cells

Nobumichi Saito, Masao Toyoda*, Masumi Kondo, Makiko Abe, Noriyuki Sanechika, Moritsugu Kimura, Kaichiro Sawada, and Masafumi Fukagawa

Division of Nephrology, Endocrinology and Metabolism, Department of Medicine, Tokai University School of Medicine, 143 Shimokasuya, Isehara, Kanagawa, 259-1193, Japan

* Correspondence: m-toyoda@is.icc.u-tokai.ac.jp; Tel.: +81-463-93-1121(ext. 2490)

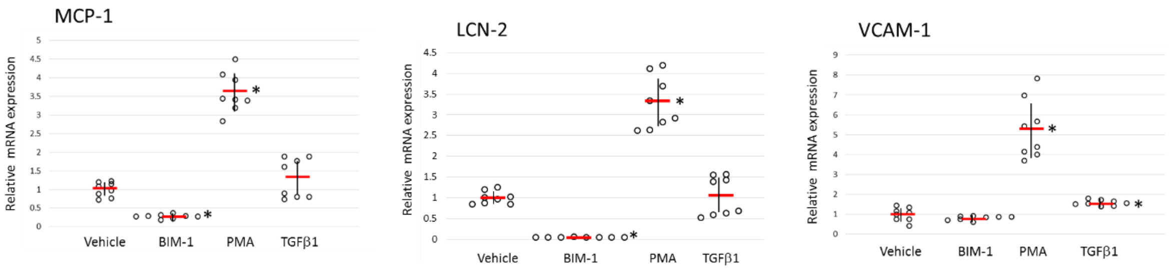

(**a**) (**b**) (**c**)

Figure S1. Effects of phosphorylation/dephosphorylation-drugs on canonical targets expressed **in a juxtaglomerular cell line (As4.1 cells).** a. Monocyte chemotactic protein 1 (MCP-1). b. Lipocalin 2 (LCN-2). c. Vascular cell adhesion molecule 1 (VCAM-1). As4.1 cells were cultured with 2 μM BIM-1, 50 ng/ml PMA and 1 ng/ml TGF-β1 for 24 h. Gene expressions were measured by qRT-PCR with TaqMan Gene Expression Assays (ThermoFisher), containing primers and probes for mouse MCP-1 (Assay ID: Mm00441242_m1), LCN-2 (Mm01324470_m1), VCAM-1 (Mm01320970_m1) and β-actin as an endogenous control. The expression relative to control (vehicle) was represented by scatter plot with the average (red bars) and S.D. (black vertical lines). Asterisks indicate a significant difference ($p<0.05$) from the control.
